# Mixture for Controlling Insecticide-Resistant Malaria Vectors

**DOI:** 10.3201/eid1411.071575

**Published:** 2008-11

**Authors:** Cédric Pennetier, Carlo Costantini, Vincent Corbel, Séverine Licciardi, Roch K. Dabiré, Bruno Lapied, Fabrice Chandre, Jean-Marc Hougard

**Affiliations:** Institut de Recherche pour le Développement, Cotonou, Bénin (C. Pennetier, S. Licciardi, F. Chandre, J.-M. Hougard); Institut de Recherche pour le Développement , Bobo-Dioulasso, Burkina Faso (C. Costantini); Centre de Recherche Entomologique de Cotonou, Cotonou (C. Pennetier, S. Licciardi, F. Chandre, J.-M. Hougard); Institut de Recherche en Sciences de la Santé, Bobo-Dioulasso (C. Costantini, R.K. Dabiré); Institut de Recherche pour le Développement, Montpellier, France (V. Corbel); Université d’Angers, Angers, France (B. Lapied); 1 Current affilations: Natural Resources Institute, Chatham Maritime, Kent, UK; and University of Sussex, Brighton, UK.

**Keywords:** Malaria, *Anopheles gambiae*, bed nets, pyrethroid, resistance, repellents, insecticides, mixtures, research

## Abstract

The spread of resistance to pyrethroids in the major Afrotropical malaria vectors *Anopheles gambiae* s.s. necessitates the development of new strategies to control resistant mosquito populations. To test the efficacy of nets treated with repellent and insecticide against susceptible and insecticide-resistant *An. gambiae* mosquito populations, we impregnated mosquito bed nets with an insect repellent mixed with a low dose of organophosphorous insecticide and tested them in a rice-growing area near Bobo-Dioulasso, Burkina Faso. During the first 2 weeks posttreatment, the mixture was as effective as deltamethrin alone and was more effective at killing *An. gambiae* that carried knockdown resistance (*kdr*) or insensitive acetylcholinesterase resistance (*Ace1^R^*) genes. The mixture seemed to not kill more susceptible genotypes for the *kdr* or *Ace1^R^* alleles. Mixing repellents and organophosphates on bed nets could be used to control insecticide-resistant malaria vectors if residual activity of the mixture is extended and safety is verified.

## Abstract 2

Impregnating mosquito nets with an insect repellent and a low dose of organophosphorous insecticide combination was effective.

Pyrethroids are the only class of insecticides that are recommended by the World Health Organization (WHO) and the Centers for Disease Control and Prevention for net impregnation to control malaria transmission (*1*,*2*). Unfortunately, malaria vector resistance to pyrethroids is becoming widespread across Africa. Pyrethroid resistance mechanisms can be divided into 2 groups: metabolic (alterations in the expression levels of activities of detoxification enzymes) and target site (nonsilent point mutations within structural receptor genes, e.g., knockdown resistance [*kdr*] mutations) (*3*). Whether the spread of resistance genes will pose a serious threat to vector control programs that are based uniquely on pyrethroid use is uncertain. Some studies have shown that *kdr* resistance does not decrease the level of protection conferred by insecticide-treated nets (ITNs) (*4*) and that ITNs do not induce *kdr* selection (*5*). Conversely, more recent studies have reported a fitness advantage for *kdr-*resistant phenotypes (*6*) and decreased efficacy of ITNs in an area of pyrethroid resistance in Benin (*7*). The need for alternative insecticidal molecules is becoming increasingly clear; however, fewer novel active ingredients are available and the timeframe needed to satisfy the regulatory requirements of public health formulations is exceedingly long (*8*). Thus, the only option for managing insecticide resistance in malaria vectors is optimal use of existing compounds. Two such tactics have already been explored: 1) the alternating use of different classes of insecticides by rotation of active ingredients and mosaic treatments (*9*,*10*), and 2) the use of insecticide mixtures (*10*,*11*).

We tested the ability of existing ingredients, a mixture of insect repellents and nonpyrethroid insecticides, to achieve vector death and excito-repellency (irritancy when mosquito contacts net and repellent activity in air) (*12*). The rationale behind this concept is that nonpyrethroid compounds can mimic the original features of pyrethroids, i.e., lethality and irritancy. Laboratory results showed that a combination of propoxur and diethyl-3-methylbenzamide (DEET) induced irritancy, knockdown, and death rates as high as those from deltamethrin against a susceptible strain of *Aedes aegypti* and significantly higher death rates against a pyrethroid-resistant strain of this mosquito. Such findings were mostly explained by a strong synergistic interaction (in terms of death and knockdown effect) between DEET and propoxur (*12*). Similar synergism was also observed in a test tunnel apparatus when DEET or another insect repellent (hydroxyethyl isobutyl piperidine carboxylate [also known as icaridin or KBR 3023]) was mixed with an organophosphate (OP; pirimiphos methyl [PM]) on mosquito nets baited with guinea pigs (*13*). These studies constitute a first step toward the development of an alternative strategy based on insect repellents for malaria vector control in areas of pyrethroid resistance.

We present results of the first evaluation of this new strategy under field conditions. The objective of the trial was to compare the efficacy of mosquito nets impregnated with mixtures of DEET+PM or KBR 3023+PM (repellent and insecticide–treated nets [RITNs]) with bed nets treated with a standard formulation of a pyrethroid (deltamethrin). The field trial was carried out in an area where *Anopheles gambiae* s.s. populations are either susceptible or resistant to pyrethroids, OPs, and carbamates, depending on the season. Seasonal influence results from temporal fluctuations in the relative frequency of the 2 molecular forms of *An. gambiae*, Mopti (M) and Savannah (S), which in this area of Burkina Faso carry insecticide-resistant alleles at contrasting frequencies (*14*,*15*). In particular, the S form carries the *kdr* and the insensitive acetylcholinesterase resistance (*Ace1^R^*) alleles at high frequency, whereas these alleles are carried at much lower frequency in the M form. This article describes the response of vector populations to the lethal effect of the formulations tested. Moreover, we investigated whether RITNs could select for the insecticide-resistance genes.

## Methods

### Study Area

The field trial was carried out during May–June and September–October 2006 in the village identified in this study as VK7, in the valley of the Kou River, near Bobo-Dioulasso, in southwestern Burkina Faso. The area is used by farmers for large-scale cultivation of rice. Throughout most of the year, rice paddies provide extensive sites for mosquito breeding, particularly of the molecular M form of *An. gambiae* s.s. Conversely, the molecular S form of this malaria vector appears mainly during the wet season, because these mosquitoes breed mostly in puddles created by rains and in other rain-dependent larval habitats.

### Insecticidal and Repellent Formulations

An OP insecticide and 2 insect-repellent formulations were evaluated as mixtures impregnated on mosquito nets. For our OP, we used Pirigrain 250 (Compagnie Générale des Insecticides, Cergy Pontoise, France), an emulsifiable concentrate formulation containing 25% PM. Our repellents were KBR 3023 and DEET. KBR 3023 was formulated as a liquid concentrate containing 25% of active ingredient. DEET was also formulated as a liquid concentrate containing 30% of active ingredient. The 2 repellent formulations are designed and distributed for application on skin by Osler (Melun, France). Deltamethrin was our pyrethroid of choice because it is one of the 2 standard pyrethroids used for net impregnation with permethrin. The water-dispersible tablets of deltamethrin were safe according WHO risk assessment and have undergone the WHO Pesticide Evaluation Scheme (*16*). For this trial, we used a standard suspension concentrate at 20% deltamethrin (Kothrin; Bayer Crop Science, Monheim am Rhein, Germany), which is routinely used to impregnate bed nets. No toxic or repellent chemicals other than those mentioned above were declared in the formulations tested.

### Mosquito Nets and Treatments

We used nets made of 100-denier polyester with a mesh size of 156 threads/square inch. To simulate the conditions of bed-net wear and tear that can be encountered in the field, 6 holes, 4 × 4 cm each, were cut on the sides and ends of each net. Three groups of nets were created: 1) nets impregnated with the repellent DEET or KBR 3023 at a dose of 10 g/m² and the insecticide PM at a dose of 150 mg/m², 2) positive-control nets dipped into standard pyrethroid deltamethrin at a dose of 25 mg/m², and 3) negative-control nets not treated.

### Experimental Huts, Volunteer Participants, and Mosquito Collections

The treated nets were set inside 4 experimental huts, according to the design and procedures described by Darriet et al. (*17*) and N’Guessan et al. (*18*). The 3.5 × 2 × 2 m huts were built with local materials and designed with 4 entry baffles that enabled mosquitoes to fly into the hut but then hindered their escape from the hut. This design enabled us to account for most mosquitoes. A veranda trap made of polyethylene sheeting and mesh screening (2 m long × 1.5 m wide × 1.5 m high) projected from the back wall of each hut. Movement of mosquitoes between the huts and the verandas was unimpeded during the night. Each hut rested on a concrete base surrounded by a water-filled moat to prevent entry of ants that would otherwise eat mosquitoes knocked down on the floor of the hut.

Local adult male volunteers were recruited to sleep on mats under the nets. They provided informed consent before enrollment. They received malaria chemoprophylaxis and medical surveillance during and 3 weeks after the trial. The Institut de Recherche pour le Développement and Burkina Faso national ethical committees formally approved the ethics of the protocol.

At 6:00 pm, before the start of the tests, the volunteers removed spiders and other mosquito predators. They then slept from 8:00 pm to 5:00 am, at which time they closed the entry baffles; lowered the curtain separating the sleeping room from the veranda-trap; and collected all mosquitoes, dead and alive, from the room, bed net, and veranda. Female mosquitoes were scored by location as dead or alive, fed or unfed; species was identified according to morphologic characteristics. To minimize bias related to mosquito attractiveness of each volunteer and spatial variation in mosquito densities, the volunteers and bed nets were rotated between huts each day. The trial was run twice, each time for 27 nights over 4 weeks. The first trial was conducted during the dry season (May 5 to June 3), when mainly the molecular M form of *An. gambiae* is present in the village; the second, during the rainy season (September 18 to October 14), when the S form predominates.

### Molecular Analyses

To determine the presence and relative frequency of the molecular M and S forms of *An. gambiae* s.s., we extracted genomic DNA from field-collected mosquitoes and amplified it by PCR according to the method of Favia et al. (*19*). The methods of Martinez-Torrez et al. (*20*) and Weill et al. (*21*) were used for molecular detection of the *kdr* and *Ace1^R^* alleles, respectively, in individual mosquitoes collected, alive or dead, from the control hut. Genotypes between live and dead mosquitoes were differentiated by using the exact test of Goudet et al. (*22*) and the software GENEPOP (*23*).

### Statistical Analysis

The effect of each treatment relative to the control was expressed in terms of the overall mosquito mortality rate ([no. immediately dead + no. dead after 24 hours]/overall no.). We considered mortality rate to have the most significant epidemiologic effect. For statistical purposes, we fitted a logistic regression model, assuming a binomial error distribution with regression parameters calculated by maximum likelihood with the software GLIM v.4 (*24*); we used the number of dead mosquitoes (*y*) as response variable, and the total number (*n*) of mosquitoes collected in the hut as binomial denominator. The proportion of dead mosquitoes (*p* = *y*/*n*) was related to time (in days) posttreatment, insecticidal treatment, and season. The statistical significance of main effects and interaction terms in the model was tested with F-tests by analysis of deviance, which involved looking at the change in deviance caused by the removal of each term from the maximal model after having allowed for overdispersion in the data by calculating a variance heterogeneity coefficient with the Williams algorithm (*25**,**26*). Median effective times (ET_50_) were calculated with the minimal model that better fits the data. Confidence limits for ET_50_ were calculated by using the Fieller theorem (*25**,**26*).

## Results

### Vector Population and Insecticide Resistance

Molecular analysis showed a marked seasonal change in molecular form composition and insecticide resistance status (Table 1). During the dry season trial, the molecular S form accounted for 5% of the *An. gambiae* s.s. population, whereas during the rainy season it represented 85%. Accordingly, the *kdr* allele, which confers resistance to pyrethroids, was found at a frequency of 8% in the *An. gambiae* s.s. sample during the dry season trial and at 88% at the end of the rainy season. Similarly, the frequency of the *Ace1^R^* allele, which confers resistance to OPs and carbamates, increased from 1% at the end of the dry season to 40% during the rainy season. The change in frequency of the insecticide resistance genes reflects the fact that these genes are carried at high frequency only in the molecular S form of *An. gambiae*.

**Table 1:** Frequency of molecular forms and alleles in *Anopheles gambiae* mosquitos, southwestern Burkina Faso*

Season	S form frequency/no. tested	*kdr* frequency/no. tested	*Ace1^R^* frequency/no. tested
May–June (dry season)	0.05/43	0.08/41	0.01/40
September–October (rainy season)	0.85/49	0.88/48	0.40/49

*Mosquito samples were randomly taken from a control (untreated) hut; S form, Savannah form; *kdr,* knockdown resistance allele; *Ace1^R^,* insensitive acetylcholinesterase resistance allele.

### Efficacy of Repellent-plus-OP Mixtures versus Deltamethrin

**Figure 1:**
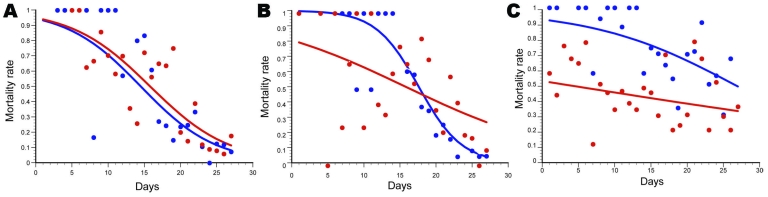
Comparative efficacy of repellent and insecticide–treated nets during dry (blue) and rainy (red) seasons. A) PM+DEET–treated nets; B) PM+KBR–treated nets; C) Kothrin–treated nets in each of 2 seasons. PM, pirimiphos methyl; DEET, diethyl-3-methylbenzamide; KBR, hydroxyethyl isobutyl piperidine carboxylate; Kothrin, 20% deltamethrin (Bayer Crop Science, Monheim am Rhein, Germany).

**Figure 2:**
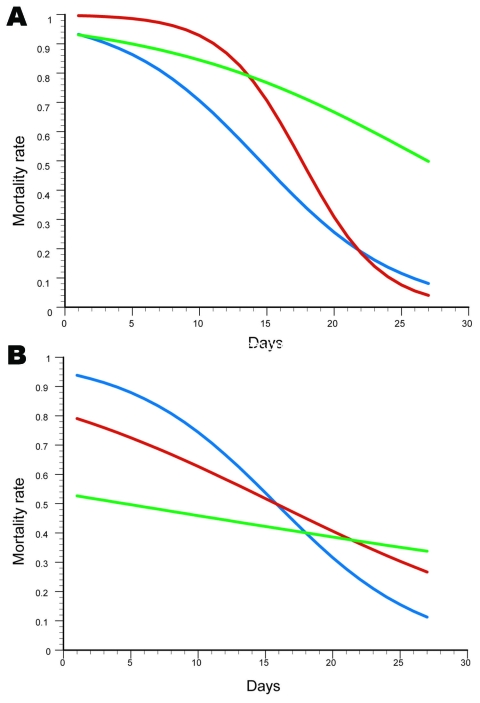
Comparative efficacy of repellent and insecticide–treated nets during A) first trial in dry season and B) second trial in rainy season. Blue lines indicate PM+DEET–treated nets; red lines indicate PM+KBR–treated nets, and green lines indicate Kothrin–treated nets. Curves drawn according to logistic plane regression of equation parameters, which are shown in Table 2. PM, pirimiphos methyl; DEET, diethyl-3-methylbenzamide; KBR, hydroxyethyl isobutyl piperidine carboxylate; Kothrin, 20% deltamethrin (Bayer Crop Science, Monheim am Rhein, Germany).

The analysis of deviance showed that the 3-way interaction term between time, treatment, and season was statistically significant (F_n,m_ = 4.705; p = 0.01), which indicates that the decrease in lethal effect over time was significantly different for treatments and between seasons. Hence, the minimal adequate model was that with a different curve relating the decrease in deaths with days posttreatment for each combination of treatments and seasons (Figures 1, 2). Accordingly, the estimates of the regression parameters for the 6 logistic curves are shown in Table 2, together with the inferred effective times in days posttreatment.

**Table 2:** Regression parameters (standard errors) and median and 90% effective duration of effectiveness of antimalarial vector treatments*

Treatment	a	b	ET_50_, d (95% CI)	ET_90_, d
First trial				
PM+DEET	2.814 (±0.737)	–0.194 (±0.039)	14.5 (11.1–16.4)	3.2
PM+KBR	5.932 (±0.628)	–0.337 (±0.032)	17.6 (16.8–18.2)	11.1
Kothrin	2.693 (±0.656)	–0.100 (±0.033)	26.8 (22.7–42.2)	4.9
Second trial				
PM+DEET	2.907 (±0.520)	–0.184 (±0.030)	15.8 (13.8–17.7)	3.9
PM+KBR	1.424 (±0.657)	–0.090 (±0.036)	15.9 (5.4–22.7)	–8.6
Kothrin	0.136 (±0.320)	–0.030 (±0.019)	4.5 (0–13.9)	–68.1

*First trial run in dry season (May and June); second trial run in rainy season (September and October). a, intercept; b, slope of curve; ET_50_ and ET_90,_ median and 90%, respectively, effective time of the minimal adequate regression model fitted to the experimental hut data; CI, confidence interval; PM, pirimiphos methyl; DEET, diethyl-3-methylbenzamide; KBR, hydroxyethyl isobutyl piperidine carboxylate; Kothrin, 20% deltamethrin (Bayer Crop Science, Monheim am Rhein, Germany).

During the dry season trial, lethality of the PM+ KBR 3023 mixture lasted longer than Kothrin over the first 15 days posttreatment (ET_90_^PM + KBR^ = 11.1 days vs. ET_90_^Kothrin^ = 4.9 days; Figure 2, panel **A**; Table 2). The PM+DEET mixture was as efficacious as Kothrin over only ≈4 days (Figure 2, panel **A**). The efficacy of the nets impregnated with the 2 mixtures decreased faster (*b*^PM + KBR^ = –0.337 ± 0.032; *b*^PM + DEET^ = –0.194 ± 0.039) than those impregnated with Kothrin (*b*^Kothrin^ = –0.099 ± 0.048) (Figure 2, panel **A**). This explains the shorter median effective time of the 2 mixtures.

The trend was different for the rainy season trial, because the lethality of nets impregnated with Kothrin was consistently lower than that of the dry season trial (Figure 1, panel **C**), in view of the change in molecular form composition and insecticide resistance status of the vector population (Table 1). Efficacies of the PM+DEET and PM+KBR 3023 were significantly higher than those for the deltamethrin formulation against the *An. gambiae* population carrying high frequencies of the *kdr* and *Ace1^R^* genes. Indeed, Kothrin never induced a mortality rate >55% throughout the course of the trial, whereas the PM+DEET and PM+KBR 3023 mixtures killed >90% of the exposed mosquitoes until ≈4 and ≈7 days posttreatment, respectively (Table 2; Figure 2, panel **B**).

Model estimates of the PM+DEET mixture did not differ between the 2 trials (Student *t* test *t_a_* = 0.248, p = 0.8; *t_b_* = 0.101, p = 0.92) (Figure 1, panel **A**), which indicates that the response in mortality rate over time was the same across seasons. Conversely, the PM+KBR 3023 efficacy changed significantly across the 2 trials (t*_a_* = 3.34, p<0.01; t*_b_* = 2.01, p<0.05); induced deaths were lower during the rainy season shortly after impregnation of the nets, but the decrease in efficacy over time was subsequently slower (Figure 2, panel **A**). Similarly, a significant difference in efficacy between the 2 seasons was observed for Kothrin (t*_a_* = 2.55, p<0.05; t*_b_* = 4.06, p<0.005); lethality was much lower during the rainy season than during the dry season; lethality of the nets, however, was always higher during the dry season trial up until the end of the 27-day replicate trials, despite a slower decrease in efficacy over time for the rainy season trial (Figure 1, panel **C**).

### Effect of Treatments on Insecticide-Resistance Genotypes

A total of 192 *An. gambiae* females were genotyped for molecular form status, *kdr* and *Ace1^R^* genes. Because of severe restrictions in gene flow between the M and S molecular forms (*27**,**28*), which led to marked differences in their resistance status (*15*), we chose to investigate the selection pressure of our 3 treatments against only the S molecular form of *An. gambiae* (88% of the total population collected during the rainy season, Table 1). The results of *kdr* genotyping of 152 specimens are shown in Table 3. The gene was in Hardy-Weinberg equilibrium (p = 1). No evidence of a significantly higher frequency of *kdr* allele was found in those that survived the 3 treatments. No S/S (susceptible homozygote) or R/S (susceptible heterozygoye) mosquito survived with the Kothrin treatments in contrast with PM+DEET and PM+KBR treatments, but susceptible genotypes were too rare to conclude about the effect of treatments on *kdr* selection pressure. The results of the *Ace1^R^* genotyping of the 153 S form of *An. gambiae* are shown in Table 3. We observed a heterozygote excess for *Ace1^R^* gene (χ² = 85.3, degrees of freedom = 8, p<0.001). No evidence of a significantly higher frequency of *Ace1^R^* allele was found in those that survived the 3 treatments.

**Table 3:** Comparative frequencies of 2 resistance genes between mosquitoes after exposure to treated nets*

Treatment	*kdr* frequency/no. tested					*Ace1^R^* frequency/no. tested			
	Surviving mosquitoes	Dead mosquitoes	p value	Total no. tested		Surviving mosquitoes	Dead mosquitoes	p value	Total no. tested
PM+DEET	0.93/22	0.98/27	0.32	49		0.50/23	0.45/28	0.51	51
PM+KBR	0.89/23	0.96/28	0.22	51		0.43/23	0.46/26	0.74	49
Kothrin	1.00/33	0.95/19	0.13	52		0.44/34	0.39/19	0.49	53

*kdr, knockdown resistance allele; *Ace1^R^,* insensitive acetylcholinesterase resistance allele; PM, pirimiphos methyl; DEET, diethyl-3-methylbenzamide; KBR, hydroxyethyl isobutyl piperidine carboxylate; Kothrin, 20% deltamethrin (Bayer Crop Science, Monheim am Rhein, Germany).

## Discussion

Our results demonstrated that a mixture of an OP (PM) and an insect repellent (either DEET or KBR 3023) on mosquito nets in an area of insecticide resistance near Bobo-Dioulasso, Burkina Faso, was as lethal as the pyrethroid deltamethrin alone for a few days against susceptible *An. gambiae* s.s. mosquitoes. However, the efficacy of each mixture was substantially higher than that of deltamethrin against a multilocus-resistant *An. gambiae* population of mosquitoes (mainly composed of the molecular S form) carrying 2 resistance genes for pyrethroids and OPs/carbamates (*kdr* and *Ace1^R^*, respectively), at moderate to high frequency. The efficacy of the mixtures was due to strong synergism between the 2 active ingredients, as demonstrated in another study (C. Pennetier et al., unpub. data). Some OPs like chlorpyriphos methyl (*11*) PM (*29*) and the carbamate carbosulfan (*29**,**30*) were also recently tested on nets to verify their efficacy in terms of induced deaths against pyrethroid-resistant populations of *An. gambiae* mosquitoes and were found to be as lethal as deltamethrin, lambda cyalothrin, or permethrin. The major constraint to the use of OPs or carbamates on bed nets is their higher toxicity for humans (*9*,*31*) and the possibility that they might induce selection pressure for resistance mechanisms other than *kdr*, such *Ace1^R^* (*32*). In view of these results, the concept of mixing an insect repellent with an OP offers a potential alternative to the use of pyrethroids on mosquito nets.

Mixtures of insect repellents and OPs have several advantages. First, the addition of a repellent enables use of lower OP dosages. The recommended dose of PM to achieve an ≈100% mortality rate is 1,000 mg/m² (*29*,*33*), 6-fold the dosage that we used in our mixtures. The possibility of using insecticides with different modes of action at lower dosages than either ingredient used alone was also observed in previous studies with OP/pyrethroid mixtures (*10*,*11*). Second, the behavioral effects of pyrethroids on mosquito nets, such as irritancy (which inhibits blood feeding), that confer personal protection to the sleeper under the net are restored by the presence of the repellent in RITNs. Previous laboratory studies on repellent-plus-OP mixtures have shown that the mixtures have the same irritant effect as pyrethroids (*12*) and that they induce protection against blood feeding (*13*). Our field trial confirmed the excito-repellency of the repellent-plus-OP mixtures (C. Pennetier et al., unpub. data). Third, we could not detect statistically significant differences in the frequency of 2 important insecticide-resistance genes, *kdr* and *Ace1^R^,* among mosquitoes that survived or died after exposure to RITNs. This finding indicates that PM+DEET and PM+KBR 3023 would not select for the *Ace1^R^* allele. Unfortunately, the high *kdr* frequency among *An. gambiae* mosquitoes did not allow us to conclude anything about the effect of RITNs on *kdr* selection pressure. RITNs should now be evaluated in an area where *kdr* allelic frequency among *An. gambiae* is moderate. Nevertheless, RITNs appear to be a promising tool for controlling malaria vectors in areas of insecticide resistance.

Our results show that mosquito deaths in response to treated nets changed between seasons, depending on the combination of repellent and insecticide used. The response to the PM+DEET mixture did not change with the resistance status of the *An. gambiae* mosquitoes, whereas the efficacy of PM+KBR 3023 decreased significantly at the end of the rainy season but lasted comparatively longer than during the dry season trial. This difference may result from a difference in mode of action of the 2 insect repellents used and their interaction with the insecticide PM. Indeed, PM is an acetylcholinesterase inhibitor, and DEET has recently been shown to exert a neurotoxic effect through alteration of neuronal function and synaptic transmission (*34*). Indeed, through elevation of intracellular calcium concentration and inhibition of the acetylcholinesterase, DEET increases the release of acetylcholine in the synaptic cleft (*34*). That led to us to hypothesize a synergism between the OP and DEET resulting from the implication of presynaptic muscarinic receptors involved in the negative-feedback regulation process (*35*), which thereby modulate acetylcholine release. Because the exact mode of action of KBR 3023 is not yet known, it is probably premature to propose an explication for why its efficacy changed in response to changes in the resistance status of the vector population.

Use of RITNs in community-based vector control programs is not yet practical because of the short persistence of the lethal effect induced by the repellent-plus-OP mixture (1–2 weeks, depending on season and combination). This effect presumably results from the high vapor pressure of the repellents, which act mainly in the vapor phase and hence do not persist long enough on the net at higher than threshold concentrations. Of note, the residual killing effect activity of RITNs in the field is much lower than that found in our previous laboratory study (*13*), probably the result of different storage conditions. In the laboratory, nets were stored in aluminium paper, which may have slowed evaporation of the active ingredient; in the present study, RITNs stayed all the day in experimental huts. However, long-lasting formulations, such as resins, microcapsules, and cyclodextrins, might increase the persistence of the mixture on the net. We suggest that industry has a vital role to play in the development of such formulations. We are currently testing a microencapsulated formulation of DEET+PM; preliminary results are encouraging (data not shown).

Another factor preventing the immediate application of RITNs in the field is the lack of knowledge of the toxic properties of repellant-plus-OP mixtures. Despite the fact that the 2 repellents and PM are reported as safe products (*36*–*40*), little is known about the interaction of repellents with OPs. We used an acetylcholinesterase inhibitor with DEET, but none of our compounds was applied on the skin. The contact between the user and the active ingredients on the bed net surface would be limited compared with a skin application, and the DEET concentration we used on nets was >3-fold lower than that recommended (30% of DEET active ingredient in commercial lotions). Nevertheless, because a mixture of chemicals must be considered as a new chemical, assessing the risk of using repellent plus OP at the operational doses used to impregnate bed nets is crucial.

In summary, application of low doses of an OP plus insect repellents as mixtures on mosquito nets was as much or more lethal shortly after application than application of the pyrethroid deltamethrin against the malaria vector *An. gambiae* in an area of resistance to multiple insecticides. The recent concept of combining repellents with insecticides is still limited by the short residual effect of the treatments and the lack of toxicologic knowledge. However, this combination appears to be a potential tool warranting further development for the control of vectors and management of insecticide resistance in malaria-endemic areas.
